# Direct Conversion of Human Fibroblasts into Adipocytes Using a Novel Small Molecular Compound: Implications for Regenerative Therapy for Adipose Tissue Defects

**DOI:** 10.3390/cells10030605

**Published:** 2021-03-09

**Authors:** Yoshihiro Sowa, Tsunao Kishida, Fiona Louis, Seiji Sawai, Makoto Seki, Toshiaki Numajiri, Kenji Takahashi, Osam Mazda

**Affiliations:** 1 Departments of Plastic and Reconstructive Surgery, Graduate School of Medical Sciences, Kyoto Prefectural University of Medicine, Kyoto 602-8566, Japan; prs-bin@koto.kpu-m.ac.jp; 2 Immunology, Graduate School of Medical Sciences, Kyoto Prefectural University of Medicine, Kyoto 602-8566, Japan; tsunao@koto.kpu-m.ac.jp (T.K.); omazda@gmail.com (O.M.); 3 Department of Applied Chemistry, Graduate School of Engineering, Osaka University, Suita, Osaka 565-0871, Japan; f-louis@chem.eng.osaka-u.ac.jp; 4 Orthopaedics Graduate School of Medical Sciences, Kyoto Prefectural University of Medicine, Kyoto 602-8566, Japan; ssawai@koto.kpu-m.ac.jp (S.S.); takahashi.b8@tohoku.ac.jp (K.T.); 5 CellAxia Inc, Nihonbashi, Tokyo 103-0012, Japan; yuki.takahashi.b8@tohoku.ac.jp

**Keywords:** adipocyte, direct conversion, fibroblast, chemical compound, adipose tissue atrophy, soft tissue reconstruction

## Abstract

There is a need in plastic surgery to prepare autologous adipocytes that can be transplanted in patients to reconstruct soft tissue defects caused by tumor resection, including breast cancer, and by trauma and other diseases. Direct conversion of somatic cells into adipocytes may allow sufficient functional adipocytes to be obtained for use in regeneration therapy. Chemical libraries of 10,800 molecules were screened for the ability to induce lipid accumulation in human dermal fibroblasts (HDFs) in culture. Chemical compound-mediated directly converted adipocytes (CCCAs) were characterized by lipid staining, immunostaining, and qRT-PCR, and were also tested for adipokine secretion and glucose uptake. CCCAs were also implanted into mice to examine their distribution in vivo. STK287794 was identified as a small molecule that induced the accumulation of lipid droplets in HDFs. CCCAs expressed adipocyte-related genes, secreted adiponectin and leptin, and abundantly incorporated glucose. After implantation in mice, CCCAs resided in granulation tissue and remained adipose-like. HDFs were successfully converted into adipocytes by adding a single chemical compound, STK287794. C/EBPα and PPARγ were upregulated in STK287794-treated cells, which strongly suggests involvement of these adipocyte-related transcription factors in the chemical direct conversion. Our method may be useful for the preparation of autogenous adipocytes for transplantation therapy for soft tissue defects and fat tissue atrophy.

## 1. Introduction

For the past 20 years, plastic surgeons have used adipose tissue as a filler for reconstruction of subcutaneous tissue defects caused by resection of tumors such as breast cancer, trauma, and other diseases [1,2,3,4]. However, adverse events may occur due to the collection of ectopic fat tissue for use as donor cells, and it is not always possible to obtain sufficient autologous fat tissue safely to achieve good functional and cosmetic results. Patients with fat tissue atrophy may also show symptoms due to lack or reduction of adipocyte functions such as energy storage, cushioning of organs and thermoregulation, as well as endocrine and secretory functions [5,6]. Adipokines secreted from adipocytes play crucial roles in physiological responses, such as metabolic homeostasis, angiogenesis, and inflammatory and immune regulation [7]. In this context, it is important to establish a procedure to prepare transplantable adipocytes from patients.

A variety of cell transplantation and tissue engineering procedures have been used for wound healing. There is considerable interest in technologies to prepare functional mature adipocytes for in vitro models for metabolic and pharmaceutical assays and for reconstructive surgery. However, long-term maintenance of mature adipocytes in culture is quite difficult, and thus, application of cultured mature adipocytes to transplantation therapy has rarely been attempted [8], although dedifferentiation and proliferation of adipocytes harvested from human fat tissues have been described [9]. Therefore, a method in which human adipocytes are induced from different cell sources, expanded in culture, and used in transplantation therapy may provide a novel and effective therapeutic modality for plastic surgery on fat tissue.

Tissue cells such as cardiomyocytes, osteoblasts, Schwann cells, and brown adipocytes can be induced from fibroblasts by transducing transcription factor genes that play crucial roles in the differentiation of the destination cells [10,11,12,13,14]. Such so-called direct reprogramming or direct conversion may produce suitable cells for transplantation. Fibroblasts can be obtained from patients with minimal invasiveness and expanded to a sufficient number in culture. Instead of transducing transcription factor genes, culturing of fibroblasts in the presence of certain chemical compounds may also induce direct reprogramming [15,16,17,18,19,20]. These transgene-free procedures may be more appropriate for cell processing for regenerative therapy compared with gene transduction because the cells induced may be less tumorigenic due to the absence of genetic aberrations associated with gene transduction. Genetically modified cells could potentially form a tumor after transplantation, because endogenous oncogenes could be activated by the vector sequence integrated into the chromosome [21]. In this study, we devised a method to convert human fibroblasts into adipocyte lineage cells by treating the cells with chemical compounds without the transfer of an exogenous gene.

## 2. Materials and Methods

### 2.1. Chemical Libraries and Compounds

Chemical libraries containing 1280 and 9600 compounds were kindly provided by the Drug Discovery Initiative, the University of Tokyo. STK287794 (2-(biphenyl-4-yloxy)-N’-[(E)-(4- hydroxy-3,5-dimethoxyphenyl)methylidene]acetohydrazide) was purchased from Vitas-M Chemical Limited(Causeway Bay, Hong Kong, China).

### 2.2. Culture Media

The complete medium consisted of Dulbecco’s modified essential medium (DMEM) (high glucose) (Invitrogen, Carlsbad, CA) supplemented with 10% fetal bovine serum (FBS), 0.1 mM non-essential amino acids (Invitrogen), 100 U/mL penicillin and 100 μg/mL streptomycin. Adipogenic medium included DMEM (high glucose) supplemented with 10% FBS, non-essential amino acids, 1 mM sodium pyruvate, 100 U/mL penicillin, 100 μg/mL streptomycin, 170 nM insulin, 1 nM 3,3′,5′-triiodo-L-thyronine, 1 μM rosiglitazone, 0.5 mM IBMX, 62.5 nM indomethacin, and 1 μM dexamethasone. The induction medium consisted of DMEM (high glucose) supplemented with 10% FBS, non-essential amino acids, 1 mM sodium pyruvate, 100 U/mL penicillin, 100 μg/mL streptomycin, 170 nM insulin, 0.5 mM IBMX, and 1 μM dexamethasone.

### 2.3. Cells

Human dermal fibroblasts (HDFs) were purchased from ScienCell Research Laboratories (Carlsbad, CA) and cultured in complete medium at 37 °C in 5% CO_2_/95% humified air (standard conditions). GFP-labeled cells were established by transducing HDFs with pMXS-GFP retroviral vector. Adipose derived stem cells (ADSCs) were isolated from aspirated fat tissue of healthy female donors and maintained in complete medium as described previously [22] (approved by the institutional ethical committee; ERB-C-487-1). To induce differentiation into adipocytes, ADSCs were cultured in complete medium under standard conditions for 4 days. When cells reached confluence, the medium was replaced by adipogenic medium. The differentiated adipocytes are referred to as “dAdipo” cells. Induced pluripotent stem (iPS) cells (253G), a kind gift from S. Yamanaka (Kyoto University), were cultured in ReproFF medium (ReproCELL, Beltsville, MD, USA) supplemented with b-FGF on SNL feeder cells derived from STO (Sandos inbred mouse (SIM) embryo-derived, 6-thioguanine-resistant, and ouabain-resistant) cells transduced with the neomycin-resistant gene and LIF gene.

### 2.4. Retroviral Vectors

Plat-GP cells were seeded in 100-mm dishes at 5 × 10^6^/dish and cultured under standard conditions. Cells were transfected with 5.0 μg of a pMxs.C/EBPβ, pMxs.c-myc [14], or pMxs.GFP (a kind gift from Professor Yamanaka at CiRA, Kyoto University) in combination with 2.5 μg of pCMV-VSV-G using the X-tremeGENE9 DNA Transfection Reagent (Roche Diagnostics, Mannheim, Germany). Cells were cultured for 24 h and the culture supernatant was replaced by fresh antibiotic-free complete medium. After culturing for another 24 h, the culture supernatant was harvested and filtered through a 0.45-μm filter. Hexadimethrine bromide was added to the viral suspension at 4 μg/μL.

### 2.5. Primary Screening

**Figure 1:**
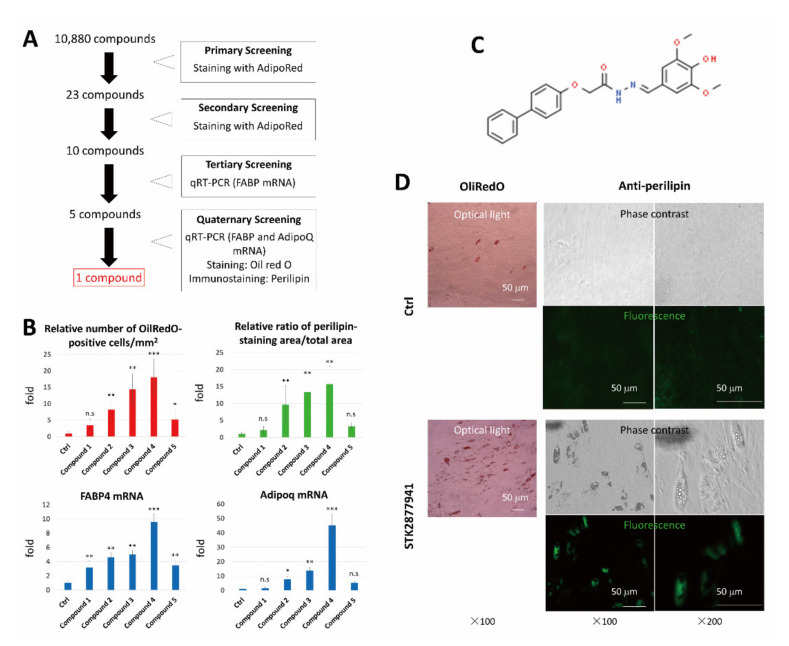
Identification of a small molecule that induces an adipocyte phenotype in human dermal fibroblasts (HDFs). (**A**) Strategy to identify compounds that induced conversion of HDFs into adipocytes. (**B**) In tertiary screening, HDFs were cultured for 14 days with the indicated compounds at 10 μM or without a compound as a control. Cells were stained with OilRed O to determine the number of OilRed O-positive cells/mm^2^ (upper left) and the ratio of perilipin-stained area/total area (upper right). RNA was subjected to real-time RT-PCR analysis to evaluate the mRNA levels of FABP4 and AdipoQ (lower). In the lower graphs, data are shown as mean ± SD normalized with respect to the β-actin mRNA level in each sample. The relative value to the control group (set to 1.0) was calculated (*n* = 6, for each group). (**C**) Structure of STK287794. (**D**) HDFs were cultured with or without 10 μM STK287794 for 14 days. Cells were stained with OilRed O or anti-perilipin antibody, followed by observation under a fluorescence microscope (middle and right). * *p* < 0.05, ** *p* < 0.01 and *** *p* < 0.001 vs. control group. ns: not significant.

HDFs were resuspended in complete medium and seeded on 96-well plates at a density of 4 × 10^3^/well (day -1). On the next day, cells were transduced with a pMxs.c-myc retroviral vector supplemented with 4 μg/μL hexadimethrine bromide. Some cells were transduced with a mixture of pMxs.C/EBPβ and pMxs.c-myc retroviral vectors and others were not transduced, as positive and negative controls, respectively. On day 1, culture supernatant was replaced by 200 μL of fresh adipogenic medium containing a single compound from the chemical libraries at a final concentration of 10 μM. On day 8, cells were washed with PBS and stained with AdipoRed Assay reagent (Lonza, Basel, Switzerland) at room temperature for 10 min. Fluorescence intensities at nine sites in each well were measured at 572 nm using a SpectraMaxM2e (BioTek Instruments, Inc., Winooski, VT, USA). Twenty-three compounds were regarded as positive and analyzed in secondary screening (Figure 1A).

### 2.6. Secondary Screening

HDFs were cultured in 96-well plates and retrovirally transduced with *c-myc* gene, as in the primary screening. Compounds were added to the cells at 5 and 10 μM in triplicate. Ten compounds that induced high fluorescence were subjected to tertiary screening.

### 2.7. Tertiary and Quaternary Screening

HDFs were seeded in 48-well plates and treated with each compound at 10 μM in triplicate, followed by RNA extraction and real time-RT-PCR analysis using probes and primers specific for human β-actin and FABP4 genes (Applied Biosystems, Waltham, MA, USA) (Supplementary Table S1). Five compounds were regarded as positive and subjected to quaternary screening. HDFs cultured in 48-well plates were treated with each of these compounds at 10 μM in triplicate, and Oil Red O (Sigma, St. Louis, MO, USA) was added to stain lipid droplets. Cells treated with the compounds were also stained with Alexa Fluor 488-labeled anti-perilipin antibody. RNA was also extracted from the cells and FABP4 and AdipoQ mRNA levels were quantified.

### 2.8. Chemical Direct Conversion

To convert HDFs into adipocytes, cells were resuspended in complete medium and seeded on 60-mm dishes at a density of 1 × 10^5^ cells/dish for ELISA, DNA microarray analysis, and transplantation experiments, or on 24-well plates at 1 × 10^4^ cells/well for Oil red O staining, qRT-PCR, and immunofluorescence (day −1). On the next day, the culture supernatant was replaced by induction medium supplemented with 1 μM STK287794. In some experiments, the culture supernatant was replaced by complete medium supplemented with 1 μM sodium pyruvate and various combinations of 1 μM STK2877941, 1 μM dexamethasone, 170 nM insulin, 0.5 μM IBMX, 62.5 nM indomethacin, 1 μM rosiglitazone, and 1 nM 3,3′,5′-triiodo-L-thyronine. The culture medium was replaced by fresh medium every 3 to 4 days. GFP-labeled chemical compound-mediated directly converted adipocytes (CCCAs) were directly converted from GFP-labeled HDFs using the same procedure.

### 2.9. Real Time RT-PCR

Total RNA was reverse-transcribed using ReverTra Ace qPCR RT Master Mix (Toyobo, Osaka, japan), and the resultant cDNA was mixed with Real-Time PCR Master Mix (Applied Biosystems, Waltham, MA) and matching probes and primers specific for human β-actin, PPARγ, SREBF1, C/EBPα, C/EBPβ, C/EBPδ, KLF15, FABP, DLK1, CD24, NANOG, and Oct3/4 (Applied Bioscience) (Supplementary Table S1). Real time PCR was carried out on a Step One Plus Real-Time PCR System (Applied Biosystems). The level (average ± S.D.) was normalized with respect to the β-actin mRNA level in each sample, and is expressed as a value relative to the control group (HDFs).

### 2.10. Immunostaining

Cells were fixed in 4% paraformaldehyde at 4 °C for 30 min. After blocking with 5% goat serum (Nacalai Tesque, Kyoto, Japan) in 0.3% Triton X-100/PBS for 1 h, the cells were incubated with rabbit anti-human perilipin antibody (1:500) (Cell Signaling Technology, Danvers, MA, USA) followed by incubation with secondary antibody conjugated with Alexa Fluor 488 (Thermo Fisher Scientific, Waltham, MA, USA). Cell nuclei were stained with DAPI (Thermo Fisher Scientific). The cells were observed under a fluorescence microscope (BZ-X710; Keyence, Osaka, Japan). BZ-II Analyzer software (Keyence, Osaka, Japan) was used to count the number of lipid droplet-positive and -negative cells to determine the ratio of adipocytes/total cells. The area occupied by the lipid droplets was also measured using the same software.

### 2.11. ELISA

Cells were cultured at 5 × 10^5^/mL for 48 h under standard conditions. The concentrations of adiponectin and leptin in the culture supernatants were measured using an ELISA Kit (R&D Systems, Minneapolis, MN). Eight measurements were performed for each assay, in which the absorbance was measured at 450 nm.

### 2.12. Glucose Uptake Assay

Glucose uptake was evaluated using a fluorescent glucose 2-NBDG (2-deoxy-2-[(7-nitro-2,1,3- benzoxadiazol-4-yl) amino]-D-glucose) Glucose Uptake Assay Kit (Cell-Based) (BioVision, Milpitas, CA: Cat. No.: #K682). Briefly, cells were seeded on a 24-well plate at 2.5 × 10^5^/400 μL and cultured overnight. Culture supernatant was replaced by fresh culture medium supplemented with 0.5% FBS. Some cells were then treated with phloretin for 45 min or left untreated, followed by the addition of 2-NBDG and Glucose Uptake Enhancer, whereas others were treated with Glucose Uptake Enhancer alone. After 30 min, cells were washed and subjected to flow cytometric analysis.

### 2.13. Surgical Procedure and Cell Implantation

All animal experiments were approved by the Committee for Animal Research, Kyoto Prefectural University of Medicine (M30–281). Care of animals was in accord with the institutional guidelines and Guide for the Care and Use of Laboratory Animals. Female NOG/BALB-Rag2 null IL-2Rγ null/NSG (NOG/SCID) mice were purchased from CLEA Japan. Cell implantation was performed as previously described with slight modifications [23,24]. Mice were anesthetized with a mixture of sublethal doses of three anesthetics (0.3 mg/kg medetomidine, 4.0 mg/kg midazolam, and 5.0 mg/kg butorphanol) (MMB). GFP-labeled CCCAs or GFP-labeled HDFs were resuspended in a 1:2 mixture of medium and Matrigel (BD Bioscience, Franklin Lakes, NJ, USA) at a density of 3 × 10^6^ cells/100 μL, and 100 μL of the cell suspension was injected into immunocompromised mice at the supraperiosteal plane of the skull using an 18-gauge needle by approaching from the posterior neck using subcutaneous tunneling to minimize graft leakage (*n* = 4 mice for each group).

### 2.14. Immunohistochemistry and Histology

At postoperative week 4, mice were euthanized with a lethal dose of isoflurane and grafts were harvested. The specimens were fixed in 4% paraformaldehyde/PBS overnight, and embedded in paraffin. The blocks were sectioned at 5 μm thickness. Immunofluorescence staining for perilipin was performed as described above. All samples were observed and analyzed with a BIOREVO microscope (BZ-9000; Keyence, Osaka, Japan) with MZ-II Analyzer software (Keyence) and an LSM510 confocal microscope (Carl Zeiss, Oberkochen, Germany). Tissue sections were also stained with HE as described elsewhere.

### 2.15. Data Analysis

Data are expressed as mean ± standard deviation (S.D.). Significance was analyzed by Student’s *t*-test and ANOVA with a Tukey–Kramer post hoc test. *p* < 0.05 was considered significant.

## 3. Results

### 3.1. Identification of a Molecule That Induces an Adipocyte Phenotype in HDFs

We previously reported that transduction of both C/EBP and c-myc genes efficiently induced conversion of HDFs into brown adipocytes, whereas this phenotypic conversion was not induced by transduction of either of the genes alone [14]. C-myc may enhance induction of the adipocyte-like phenotype in HDFs. Therefore, we hypothesized that a molecule capable of converting HDFs into adipocytes may be more efficiently detected by addition to c-myc-transduced HDFs than to non-transduced HDFs. As a rapid and high throughput assay suitable for the first screening, we stained lipid droplets in cells with fluorescent AdipoRed Assay reagent. Initial screening of 10,880 compounds (Supplementary Figure S1) yielded 23 hits (0.21%) that significantly induced accumulation of intracellular lipids in HDFs transduced with the c-myc gene (Figure 1A). The activities of the selected compounds were confirmed by secondary screening, which gave reproducible positive results for 10 compounds. These compounds were then subjected to tertiary and quaternary screening using HDFs that were not transduced with c-Myc. As shown in Figure 1B, some compounds significantly induced abundant lipid droplets, expression of perilipin, and FABP4 and AdipoQ mRNA expression. Finally, we chose one compound (“Compound 4” in Figure 1B) (Figure 1C) that most strongly induced adipocyte markers (Figure 1D), including FABP4, AdipoQ mRNA, and perilipin (Figure 1B,D).

In the first and secondary screening rounds, c-myc-transduced HDFs was used, while STK287794 converted untransduced HDFs into CCCAs. To estimate the possibility that the endogenous c-myc was induced by an addition of insulin, IBMX, and dexamethasone, which are contained in the induction medium, we evaluated expression of c-myc mRNA in HDFs cultured in complete medium or induction medium. The c-myc mRNA was not significantly elevated in HDFs cultured in the induction medium (Supplementary Figure S2), excluding the possibility that insulin, IBMX, and dexamethasone contributed to the cell conversion by inducing endogenous c-myc.

The cells treated with STK287794 were subjected to detailed analyses as representative CCCAs.

### 3.2. Determination of Suitable Culture Conditions for CCCA Induction

**Figure 2:**
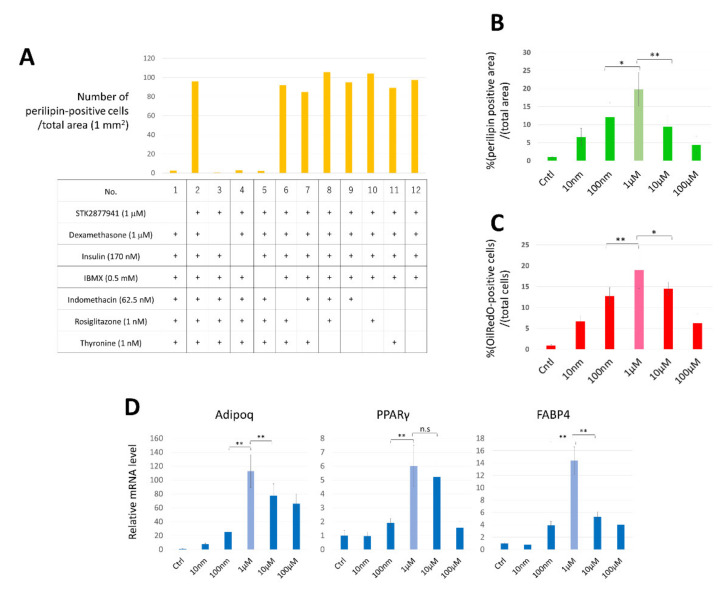
Optimization of culture conditions in which STK287794 converted HDFs to chemical compound-mediated directly converted adipocytes (CCCAs). (**A**) HDFs were cultured in complete medium supplemented with 1 mM sodium pyruvate and the indicated components for 14 days. Cells were immunostained with anti-perilipin antibody and the number of perilipin-positive cells/mm^2^ was determined. (**B**–**D**) HDFs were cultured in induction medium supplemented with the indicated concentrations of STK287794 for 14 days. Cells were stained with anti-perilipin antibody (**B**) or OilRed O (**C**), and RNA was extracted from the cells and mRNA levels for the indicated adipocyte marker genes were measured by real-time RT-PCR (**D**). Data are expressed as the mean ±.SD from sextuplicate (**A**) or triplicate (**B**–**D**) samples. Relative values to control (set to 1.0) are plotted. * *p* < 0.05, and ** *p* < 0.01 vs. control group. ns: not significant.

To determine the appropriate composition of the culture medium for direct conversion, adipogenic media deprived of a single component were supplemented with STK287794 and tested for CCCA induction. Expression of perilipin was evaluated by immunostaining. As shown in Figure 2A, removal of dexamethasone, insulin, or IBMX almost completely abolished induction of perilipin-positive cells, whereas indomethacin, rosiglitazone, and thyronine were not essential for cell conversion. Thus, we omitted indomethacin, rosiglitazone, and thyronine from the adipogenic medium, and used the resultant medium as “induction medium” in subsequent experiments.

Next, we assessed the optimal concentration of STK287794 for cell conversion. HDFs were cultured with various concentrations of STK287794 for 14 days, and expression of perilipin was evaluated by immunostaining (Figure 2B), accumulation of lipid droplets was determined by OilRed O staining (Figure 2C), and expression of mRNAs for PPARδ, Fabp4, and Adipoq was measured (Figure 2D). A concentration of 1 μM was found to be optimal based on all indexes. OilRed O-positive cells accounted for approximately 19.0% of the cells cultured with 1 μM STK287794 (Figure 1D), indicating an efficiency of direct conversion of approximately 19.0%.

### 3.3. Upregulation of C/EBPα and PPARγ during Conversion of HDFs into CCCAs

**Figure 3:**
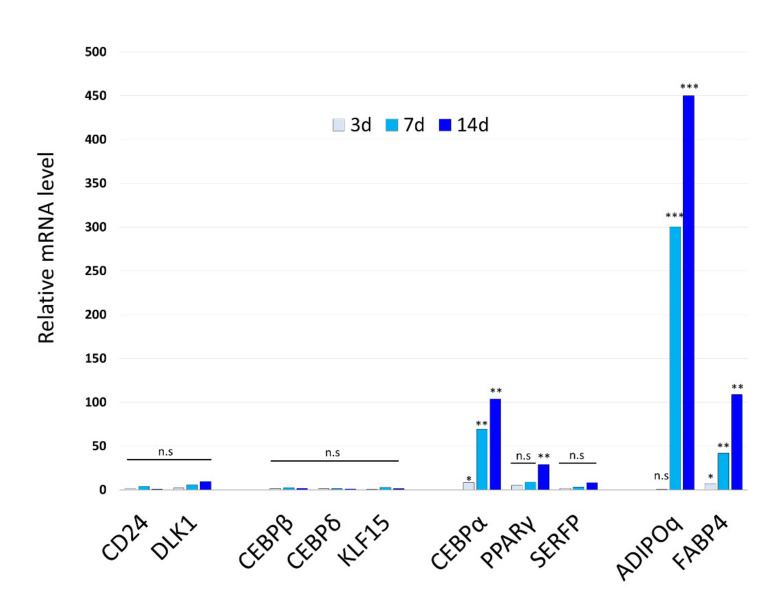
Adipogenic marker genes are induced in HDFs by STK287794. HDFs were cultured in induction medium with or without 1 μM STK287794. After the indicated number of days, RNA was extracted from the cells and mRNA expression levels for the indicated genes were determined by real-time RT-PCR. Values (mean ± SD) are normalized with respect to the β-actin mRNA level in each sample. Relative values to control (cells cultured without STK287794 for the same period; set to 1.0) are shown (*n* = 3 for each group). *p < 0.05, **p < 0.01, ***p < 0.001 vs. control group. n.s: not significant.

To evaluate expression of adipocyte-related genes in CCCAs, expression of mRNAs for fat marker and transcription factor genes was examined by real-time RT-RCR at different time points during cell conversion. As shown in Figure 3, *PPARγ*, *C/EBPα*, Fabp4, and *Adipoq* mRNAs were significantly increased upon induction, whereas expression of *C/EBPβ* and *C/EBPδ* did not change markedly (Figure 3). Expression of Adipoq and Fabp4 was increased by 100 times or more, whereas expression of brown/beige adipocyte markers, DIO2 and PRDM16, was not significantly elevated in CCCAs (Supplementary Figure S3). Among transcription factors, C/EBPα was upregulated in HDFs as early as 7 days after exposure to STK287794, and C/EBPα and PPARγ were both elevated on day 14. These findings suggest that C/EBPα may be induced by STK287794 treatment and play an important role in conversion of HDFs into adipocytes by affecting expression of other adipocyte-related transcription factors, including PPARγ.

### 3.4. Absence of a Pluripotent Stem Cell-Like State during Conversion of HDFs into CCCAs

**Figure 4:**
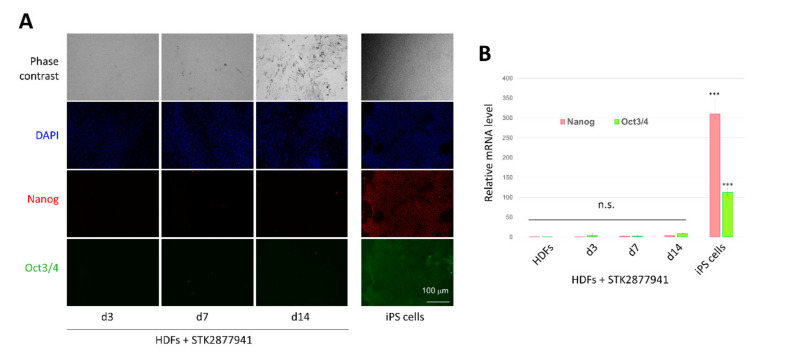
A pluripotent state does not occur during conversion from HDFs to CCCAs. HDFs were cultured with STK287794 as in Figure 3 for the indicated number of days. (**A**) Cells were stained with anti-Nanog and anti-Oct3/4 antibodies, and nuclear staining was performed with DAPI. As a positive control, human iPS cells were also stained. Fluorescence microscopic images are shown (original magnification: ×100). (**B**) RNA was extracted from the cells and subjected to real-time RT-PCR to evaluate Nanog and Oct3/4 mRNA levels. Values (mean ± SD) are normalized with respect to the β-actin mRNA level in each sample. Relative values to the control were calculated (*n* = 3 for each group). *** *p* < 0.001 vs. control group. n.s: not significant.

To exclude the possibility that CCCAs were indirectly induced from HDFs by passing through a pluripotent stem-cell-like state, we examined the expression of pluripotent stem cell marker genes during cell conversion. Neither Nanog nor Oct3/4 were significantly detected on days 3, 7, and 14 after addition of STK287794, as revealed by immunostaining (Figure 4A) and real-time RT-PCR (Figure 4B). These results strongly suggest that HDFs were directly converted to CCCAs.

### 3.5. CCCAs Share Similar Functional Properties with Mature Adipocytes In Vitro

**Figure 5:**
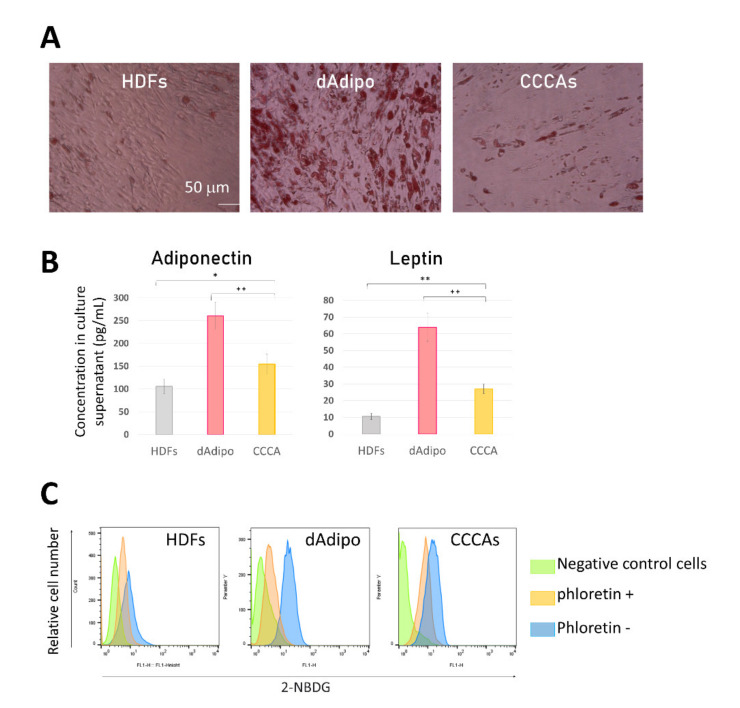
CCCAs have mature adipocyte-like functions in vitro. (**A**) HDFs, differentiated adipocytes (dAdipo), and CCCAs were stained with OilRed O. Optical light microscopic images are shown (original magnification: ×100). (**B**) Culture supernatants of the indicated cells were subjected to ELISA for measurement of concentrations of the indicated adipokines. (**C**) Cells were or were not pretreated with phloretin and were then tested for incorporation of 2-NBDG in the presence or absence of Glucose Uptake Enhancer. Cells were analyzed by means of flow cytometry. Representative histograms for green fluorescence are shown. * *p* < 0.01, ** *p* < 0.001 vs. control group; ^++^*p* < 0.01 vs. dAdipo group.

Next, the functional characteristics of CCCAs were assessed against positive control human ADSCs that were induced to differentiate into adipocytes (dAdipo) that were rich in OilRed O-positive lipid droplets (Figure 5A). ELISAs were used to measure adipokine levels in the supernatants of HDFs, dAdipo, and CCCAs. The CCCAs secreted adiponectin and leptin at significantly higher levels compared to HDFs, although dAdipo secreted even more of these adipokines (Figure 5B).

Glucose uptake in CCCAs was also analyzed. This uptake was higher in CCCAs than in HDFs, and similar to that in dAdipo (Figure 5C). Addition of phloretin, an inhibitor of sodium-glucose linked transporter 1 (SGLT1) and SGLT2, slightly reduced glucose uptake in CCCAs, indicating partial involvement of SGLT1 and SGLT2 in this uptake. This was in contrast to the results in dAdipo, in which glucose uptake was much more dependent on SGLT1 and SGLT2.

### 3.6. CCCAs Remain Adipocyte-Like and Are Found in Granulation Tissue Formed after Implantation

**Figure 6:**
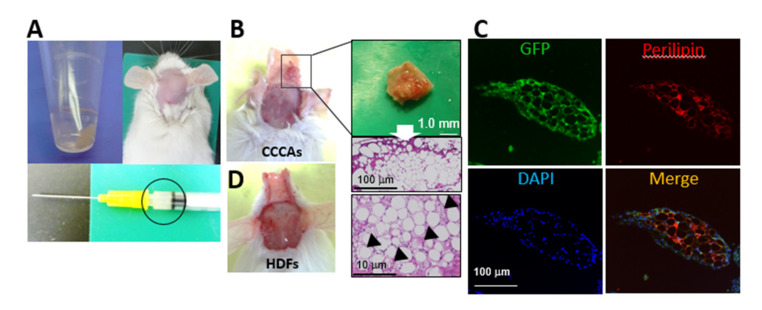
CCCAs reside in granulation tissue after subcutaneous implantation in mice. NOG/SCID mice were implanted with GFP-labeled CCCAs or GFP-labeled HDFs (**A**, upper left and lower panels) in the supraperiosteal plane of the skull (**A**, upper right) (*n* = 4 mice per group). Four weeks later granulation tissue containing fat was observed at the site implanted with GFP-labeled CCCAs (**B**). Tissue sections were subjected to HE and immunohistochemical staining using anti-perilipin antibody, and cell nuclei were also stained with DAPI. The arrowheds indicates adipocyte with multi-locular lipid droplets (**C**). No granulation tissue was found in mice implanted with GFP-labeled HDFs (**D**).

To examine whether CCCAs stay in soft tissue after implantation, we implanted GFP-labeled CCCAs and GFP-labeled HDFs as a control at the supraperiosteal plane of the skull of NOG/SCID mice (Figure 6A). Four weeks after implantation, granulation tissue containing fat was found at the implantation site (Figure 6B). The HE staining demonstrated that the tissue was mainly composed of white adipocyte-like cells with a uni-locular lipid droplet, whereas some multi-locular brown/beige adipocyte-like cells were also detected (Figure 6B). Immunohistochemical analysis showed that the GFP signal colocalized with perilipin expression in the granulation tissue (Figure 6C), indicating that the implanted CCCAs had survived in this tissue without undergoing necrosis, and continued to have an adipocyte phenotype in the implanted subcutaneous region. In contrast, implantation of GFP-labeled HDFs did not result in the formation of this granulation tissue (Figure 6D).

## 4. Discussion

Even terminally differentiated somatic cells can be epigenetically reprogrammed, leading to phenotype conversion of the cells into a different lineage [10,11,12,13,14,15,16,17,18,19,20]. In this study, we developed a procedure to induce adipocytes from HDFs by stimulating the cells with a single small molecule, STK287794. Such a chemical-based conversion strategy allows for the generation of adipocytes without adding genetic modifications. We determined the minimally required components in the culture medium for efficient induction of conversion into CCCAs, which may be useful for future clinical applications.

The mechanisms by which STK287794 induces conversion of HDFs into CCCAs remain to be determined. Generally, direct reprogramming is triggered by key transcription factors that play crucial roles in physiological differentiation of the cell type [25]. For differentiation of preadipocytes to adipocytes, PPARγ and C/EBP family members are the key transcription factors, orchestrating transcriptional cascades to elicit adipocyte differentiation [26,27]. PPARγ and C/EBPα are regarded as late adipose differentiation markers and master regulators of adipocyte differentiation. PPARγ expressed during the metaphase is essential for adipocyte differentiation, since it enhances expression of specific lipogenic and adipogenic genes during preadipocyte differentiation and is necessary and sufficient for fat formation [28].

C/EBP-α plays important roles in the early stages of preadipocyte differentiation, and, in cooperation with PPAR-γ, activates additional adipocyte genes [29,30]. PPARγ and C/EBPα enhance expression of fatty acid synthase (FAS), which is involved in fat accumulation, and of SREBP-1, a transcription factor involved in cholesterol metabolism, resulting in fat cell hypertrophy [31,32,33,34]. Downregulation of C/EBP-α and PPAR-γ inhibits lipid accumulation and suppresses adipocyte differentiation [35,36]. PPARγ and C/EBPα are usually induced by the transcription factors C/EBPβ and C/EBPδ, which are sometimes referred to as early differentiation markers and are expressed in the transient cell growth phase in the early stage of adipocyte differentiation [27,37]. STK287794 stimulated lipid deposition but failed to influence the expression of adipogenic transcription factors C/EBPβ, C/EBPδ, and KLF15 (Figure 3). These results suggest that C/EBPα has an important role in the mechanisms of conversion from HDFs to CCCAs, whereas the involvement of early stage transcription factors is limited.

We also analyzed the expression of pluripotent stem cell marker genes during conversion from HDFs to adipocytes to exclude the possibility of a transient stem-like state. There was no expression of Nanog or Oct3/4 (Figure 5C), providing supporting evidence that the conversion process bypassed the pluripotent state.

We used HDFs derived from adult dermis as human somatic cells to be converted into adipocytes because HDFs may be suitable for use in clinical applications. HDFs have a high proliferation capacity in vitro regardless of the age of the donors [38], so our procedures may be applicable to elderly people, who have a high incidence of adipose disorders. The method offers adipocytes that may be safely transplanted in patients because the CCCAs do not contain pluripotent stem-like cells (Figure 4) and are free from risks associated with genomic modification [39]. This chemical compound-based method may enable the preparation of autologous adipocytes at relatively low cost.

Since adipocytes play essential roles in development, homeostasis, and diseases of adipose tissue [40,41], direct conversion of HDFs into adipocytes may also be useful for analysis of the functions and pathophysiology of adipocytes in vitro. Notably, there is accumulating evidence that cancer cells can be induced to differentiate into adipocytes by small molecules, which may provide new strategies for cancer treatment [42,43,44,45,46]. Further studies in the future may clarify whether STK2877941 is useful in cancer therapy by inducing malignant cells into benign adipocytes. STK2877941 may also contribute to the treatment of intractable diseases related to lipoatrophy, in which adipose tissue is systemically or partially lost. It may also be beneficial to substitute CCCAs for aberrant adipocytes in patients with diabetes, hypertriglyceridemia, and fatty liver [47].

To our knowledge, this study is the first to convert human dermal fibroblasts into functional adipocytes in vitro, although the efficiency of conversion remains unsatisfactory at present. The CCCAs stayed in granulation tissue after implantation in mice and continued to show an adipocyte-like phenotype. Further studies are needed to improve the efficiency of conversion and to advance this technology for potential regenerative therapy.

## 5. Conclusions

We screened chemical libraries and identified STK287794 as a molecule that induces an adipocyte-like phenotype in HDFs. This is the first study to show that HDFs can be converted directly into adipocyte-lineage cells without introducing an exogenous transgene. This method may help to generate autogenous adipocytes that could be used for transplantation, paving the way for novel regenerative plastic surgery.

## Data Availability

The data presented in this study are available on request from the corresponding author. The data are not publicly available.

## Supplementary Materials

The following are available online at https://www.mdpi.com/2073-4409/10/3/605/s1. Supplementary Table S1: List of real-time RT-PCR primers and probes. Supplementary Figure S1: Results of the first screening. Fluorescence intensities of representative samples in the first screening, with samples sorted in descending order. Screening was performed as described in the text. Supplementary Figure S2. c-myc expression was not affected by insulin, IBMX, and dexamethasone. HDFs were cultured in complete medium lacking insulin, IBMX, and dexamethasone or in induction medium containing insulin, IBMX, and dexamethasone. Three days later RNA was extracted and c-myc mRNA level was analyzed by means of real-time RT-RCR. n.s: not significant. Supplementary Figure S3. CCCAs did not significantly express brown/beige adipocyte markers. HDFs were cultured in induction medium with or without 1 μM STK287794. After 14 days, RNA was extracted from the cells and mRNA for the indicated genes was evaluated by means of real-time RT-PCR. Value (mean ± SD) is normalized with respect to the β-actin mRNA level in each sample. Relative values to control (cells cultured without STK287794; set to 1.0) are shown (*n* = 6 for each group). n.s: not significant.
