# Supplementary material for: Direct Conversion of Human Fibroblasts into Adipocytes Using a Novel Small Molecular Compound: Implications for Regenerative Therapy for Adipose Tissue Defects
Source: Cells. 2021 Mar 9;10(3):605. doi: 10.3390/cells10030605 (PMC8000077; doi:10.3390/cells10030605)
Supplement: Supplementary file 1 [file cells-10-00605-s001.pdf]

Supplementary Table S1

Supplementary Table S1 List of real-time RT-PCR primers and probes

Target	Description/Sequences
hβ-actin	Applied Bioscience Hs01060665_g1
hSREBF1	Applied Bioscience Hs01088691_m1
hC/EBPα	Applied Bioscience Hs00269972_s1
hC/EBPβ	Applied Bioscience Hs00942496_s1
hC/EBPδ	Applied Bioscience Hs00270931_s1
hKLF15	Applied Bioscience Hs00362736_m1
hFABP	Applied Bioscience Hs01086177_m1
hAdipoq	Applied Bioscience Hs00605917_m1
hDLK1	Applied Bioscience Hs00171584_m1
hCD24	Applied Bioscience Hs00909233_m1
hNANOG	Applied Bioscience Hs03044178_g1
hOct3/4	Applied Bioscience Hs00999634_g1
hDIO2	Applied Bioscience Hs00988260_m1
hPRDM16	Applied Bioscience Hs00223161_m1
hKCNK3	Applied Bioscience Hs00605529_m1
hC-MYC	Applied Bioscience Hs00153408_m1
hUCP1	Forward: 5’-AAGTGTGCCCAACTGTGCAAT-3’
	Reverse: 5’-TGACGTTCCAGGATCCAAGTC-3’

Supplementary Figure S1

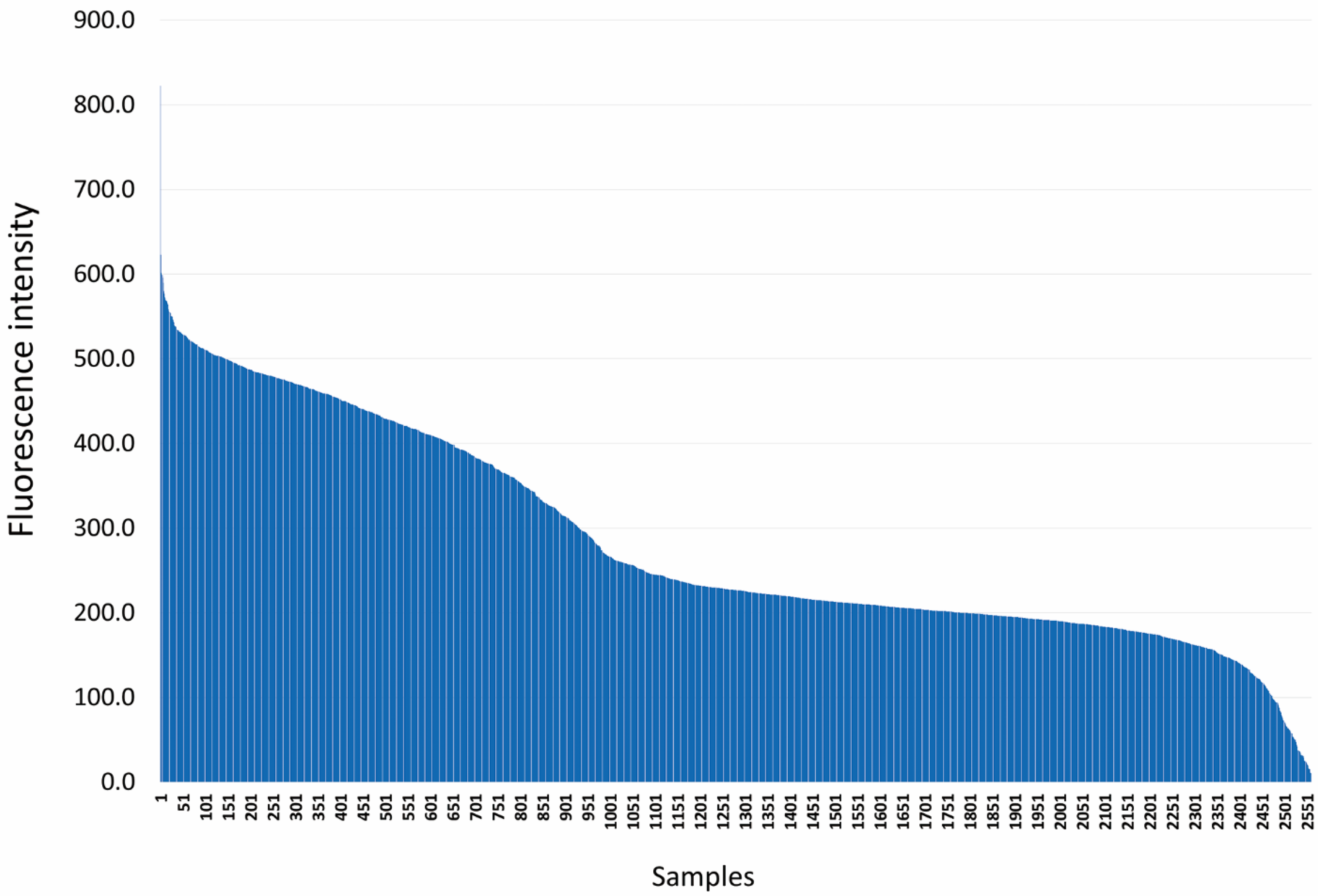

Supplementary Fig. S1
First screening of the chemical compounds were performed as described in the text. Fluorescence intensities of representative samples are shown, in such a manner that the samples are sorted in descending order.

Supplementary Figure S2

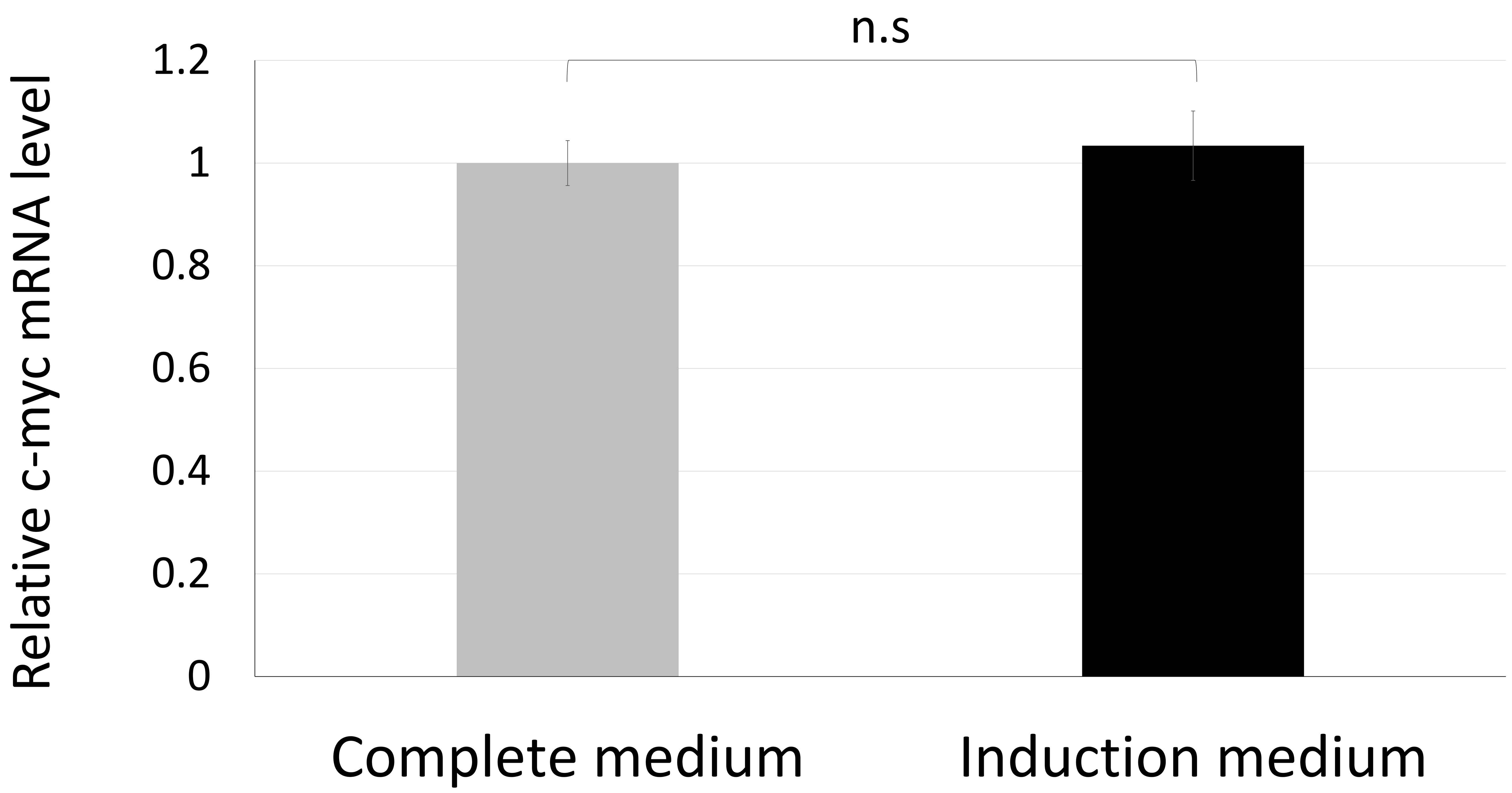

Supplementary Fig. S2.
c-myc expression was not affected by insulin, IBMX and dexamethasone. HDFs were cultured in complete medium lacking insulin, IBMX and dexamethasone or in induction medium containing insulin, IBMX and dexamethasone. Three days later RNA was extracted and c-myc mRNA level was analyzed by real-time RT-RCR. n.s: not significant.

Supplementary Figure S3

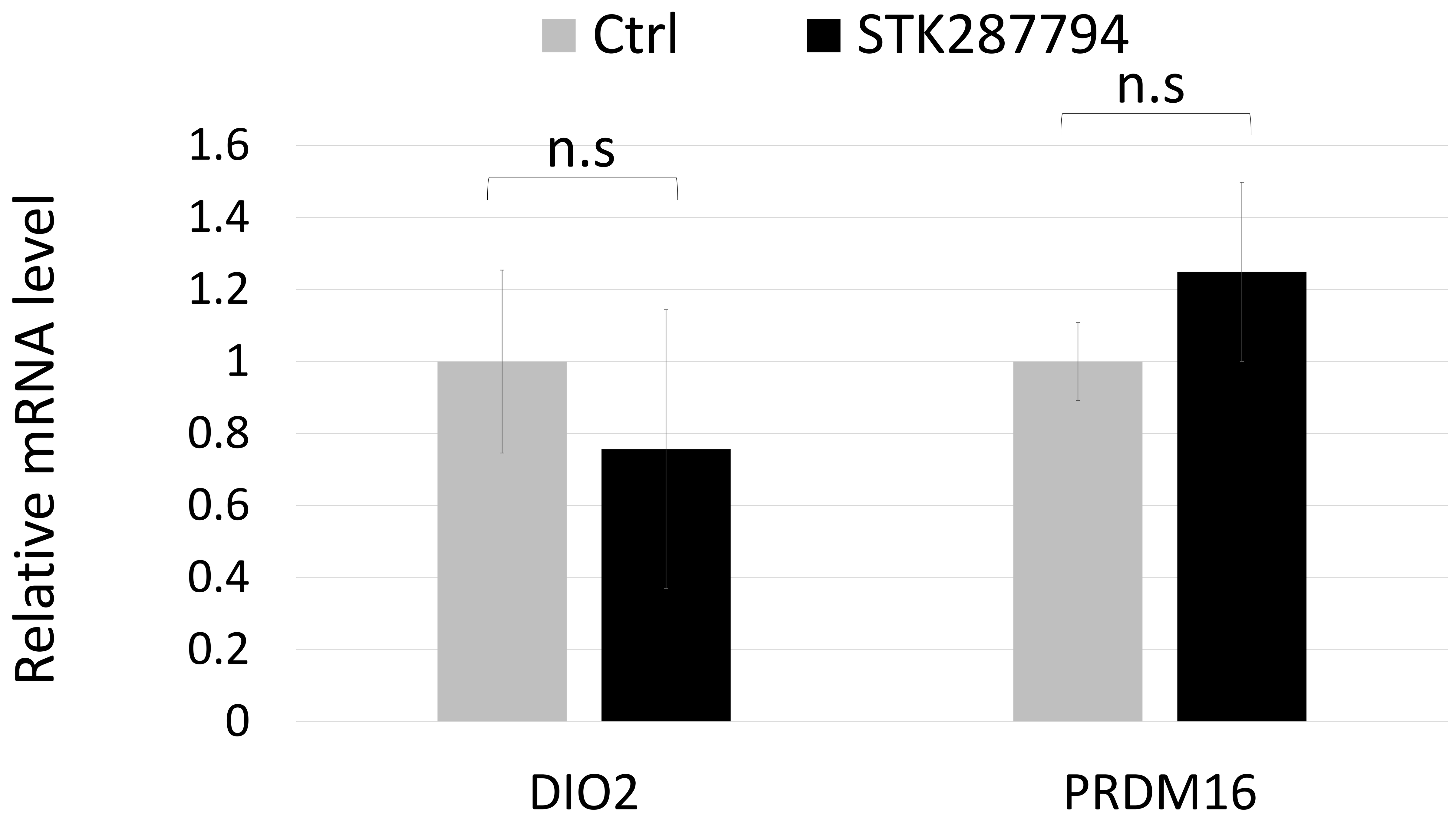

Supplementary Fig. S3
CCCAs did not significantly express brown/beige adipocyte markers. HDFs were cultured in Induction Medium with or without 1 µM STK287794. After 14 days, RNA was extracted from the cells and mRNA for the indicated genes was evaluated by real-time RT-PCR. Value (mean ± SD) is normalized with respect to the β-actin mRNA level in each sample. Relative values to control (cells cultured without STK287794; set to 1.0) are shown (n=6 for each group). n.s: not significant.
